# Intravitreal cysticercosis presenting as neovascular glaucoma

**DOI:** 10.4103/0301-4738.58478

**Published:** 2010

**Authors:** Dhanashree Ratra, Chekitaan Phogat, Maneesh Singh, Nikhil S Choudhari

**Affiliations:** Bhagawan Mahavir Vitreoretinal Services and Department of Glaucoma, Sankara Nethralaya, Chennai, India

**Keywords:** Bevacizumab, cysticercosis, neovascular glaucoma, vitrectomy

## Abstract

We report two cases of intraocular cysticercosis which showed a peculiar presentation of neovascular glaucoma which is hitherto unreported. Two young adults presented with symptoms of raised intraocular pressure due to neovascular glaucoma. On dilated fundus examination both were found to have dead intravitreal cysticercosis. The cysts were removed by a three-port vitrectomy and intracameral injection of bevacizumab was given to help in the regression of rubeosis. Trabeculectomy had to be combined in one case. The intraocular pressure returned to normal. No recurrence of rubeosis was seen even after one year.

## Introduction

Ocular cysticercosis affects various tissues of the eye such as the extraocular muscles, subretinal space and the vitreous. The intraocular location of the cyst causes retinal detachment, macular scarring,[1,2] retinal vasculitis and vitritis.[3] Only one case report describes cysticercosis resulting in glaucoma due to pupillary block.[4] We report here two cases of intraocular cysticercosis, which presented with neovascular glaucoma (NVG). To the best of our knowledge this is the first report of NVG due to intraocular cysticercosis.

## Case Reports

### Case 1

**Figure 1:**
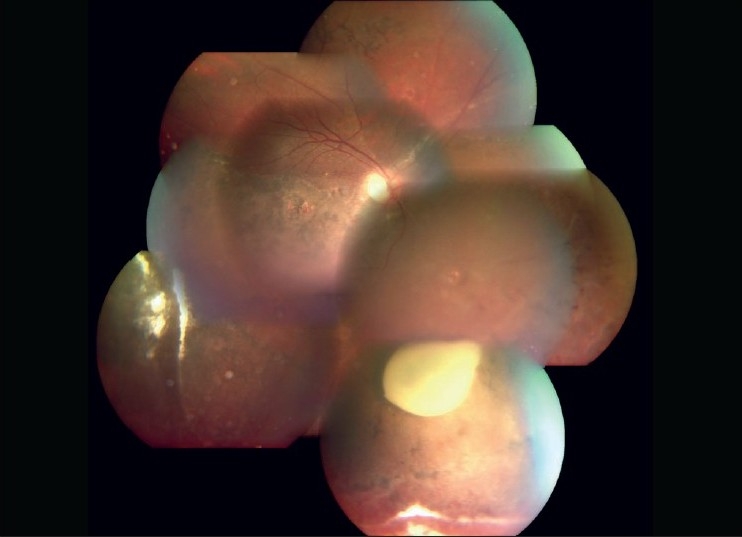
Color montage photograph of the right fundus of the patient showing whitish cystic lesion in the inferior quadrant

**Figure 2:**
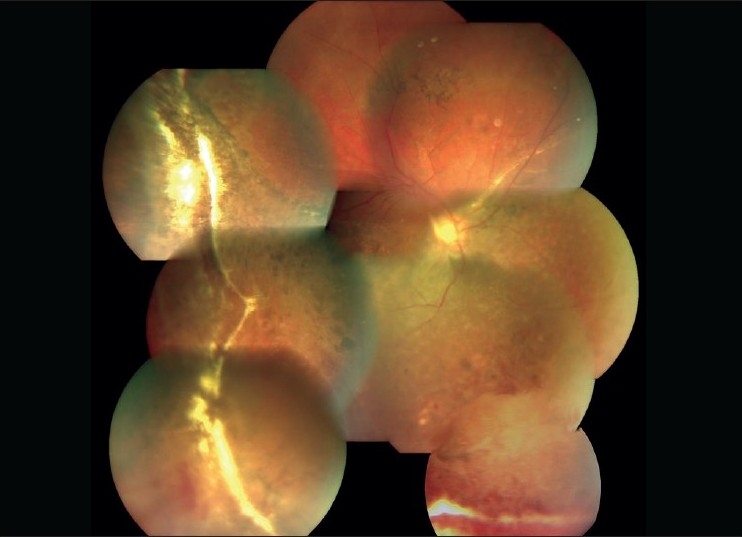
Postoperative fundus photo of Case 1 showing clear vitreous cavity with attached retina

A 26-year-old male, vegetarian by diet, presented with a history of painful decrease in vision of the right eye since 15 days. He had undergone scleral buckling five years ago and was maintaining stable vision. The left eye had no light perception following long standing total retinal detachment. His best-corrected visual acuity in the right eye was 20/200. Slit-lamp biomicroscopy revealed 360-degree florid rubeosis, 1+ aqueous cells and flare. Intraocular pressure (IOP) was 42 mm Hg. Gonioscopy showed open angle and 360 degrees neovascularization. Fundus examination revealed attached retina with peripheral buckle effect. The inferior retina showed pigmentary changes comitant with spontaneous reattachment. There was no evidence of any vascular occlusion. In addition an elongated cyst was seen projecting into the vitreous cavity adherent to the retina in the inferonasal quadrant of the fundus [Fig. 1]. Mild optic disc hyperemia was noted. There was no movement of the cyst on examination and no scolex was seen even on ultrasound examination. Based on the above findings a clinical diagnosis of a dead ocular cysticercus cyst with inflammatory response and secondary NVG was made. Immediate medical treatment was started with tab acetazolamide 250 mg four times daily, topical timolol maleate twice daily and topical brimonidine thrice daily to control the IOP. With the treatment the IOP reduced to 20 mm Hg. The patient then underwent a three-port pars plana vitrectomy. Retinal attachments of the cyst were cut. The cyst was lifted into the vitreous cavity with a backflush needle and removed intact through sclerotomy. Mild scatter laser was applied to the site of attachment. Bevacizumab (Avastin) 0.05 ml (1.25 mg) was injected intracamerally with a 30-G needle. Degenerated cysticercus was confirmed on histopathologic evaluation. After a week, there was complete regression of rubeosis with quiet anterior chamber. The IOP was controlled with topical dorzolamide and brimonidine drops. Fundus examination showed attached retina and normal optic disc [Fig. 2]. Gonioscopy done two weeks later revealed complete regression of the angle neovascularization. After one year, the patient is maintaining 20/160 vision in the right eye with IOP of 18 mm Hg (on two medications) and no recurrence of iris or angle neovascularization.

### Case 2

**Figure 3:**
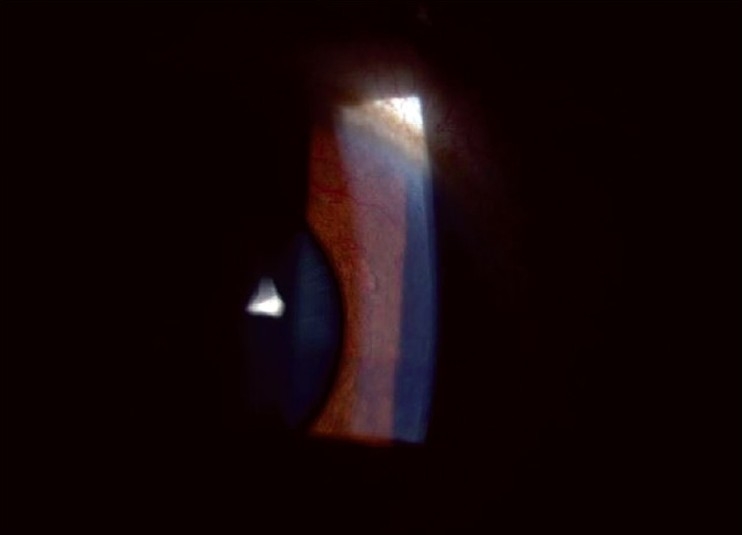
Preoperative slit-lamp photograph of left eye of Case 2 demonstrating rubeosis iridis

**Figure 4:**
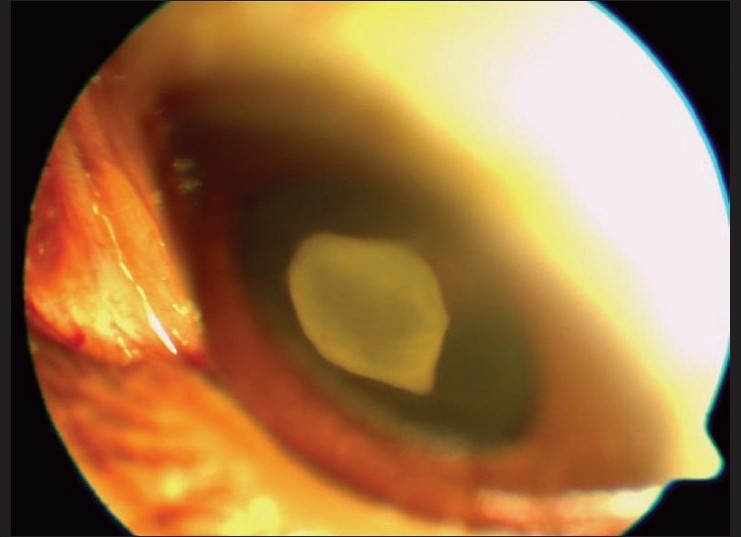
Slit-lamp photo of Case 2 showing cysticercus cyst in the anterior vitreous cavity

An 18-year-old student, non-vegetarian by diet, presented to us with complaints of episodes of decreased vision, redness and haloes in the left eye, since two months. On examination his best-corrected visual acuity was 20/40 in the left eye. Slit-lamp biomicroscopy showed ciliary congestion, corneal edema, aqueous flare and 360 degree rubeosis iridis [Fig. 3]. IOP was 44 mm Hg. Angle was closed for 270 degrees due to neovascularization. Fundus examination showed a white opaque cyst in the anterior vitreous cavity [Fig. 4]. No scolex or movements of the cyst were noted. The optic nerve head showed 0.9 cupping. The retina was attached throughout with normal vessels and macula. Ultrasound B-scan confirmed absence of scolex. Humphrey visual field test (24-2) revealed advanced field loss. The right eye was normal.

**Figure 5:**
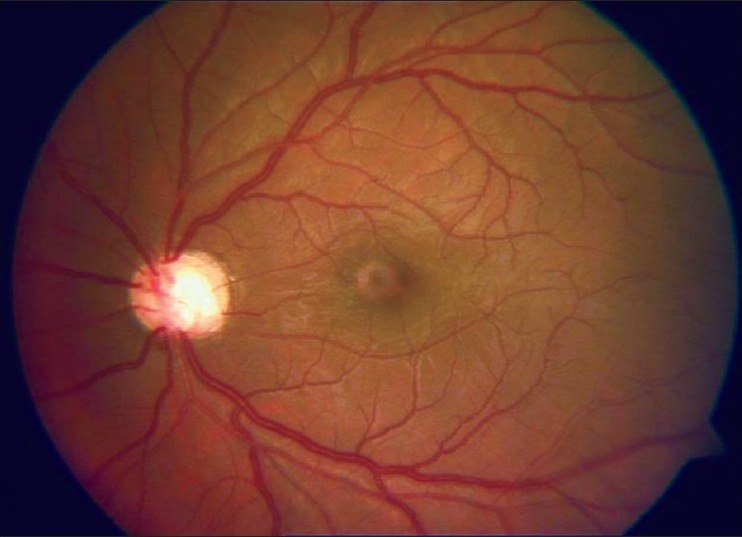
Postoperative fundus picture of Case 2. Note the optic nerve head cupping

The patient was started on oral acetazolamide 250mg four times daily, topical timolol maleate twice daily along with topical brimonidine thrice daily. He underwent a three-port pars plana vitrectomy. The cyst was freed from the surrounding vitreous and was removed intact using mild aspiration from the vitreous cutter. The vitrectomy was completed in usual fashion. A trabeculectomy with Mitomicin C was then performed and intracameral injection of 1.25 mg of bevacizumab (Avastin) was given. One week postoperatively the IOP was well controlled (13 mm Hg) with complete regression of the rubeosis. Two weeks postoperatively a localized retinal detachment was noted inferiorly with a horseshoe tear. The patient was immediately operated with fluid gas exchange with C3F8 gas and buckling of the break. The IOP remained controlled without any medications, no rubeosis was seen and retina was attached till the last visit at six months follow-up [Fig. 5].

## Discussion

Cysticercosis is a parasitic infection caused by *Cysticercus cellulosae*, the larval form of cestode, *Taenia Solium*.[1] Ocular or adnexal involvement occurs in 13–46% of patients.[3] Reportedly 35% of the cysts are in the subretinal space, 22% in the vitreous, 22% in the subconjunctival space, 5% in the anterior segment and 1% in the orbit.[3] Among the intraocular cysts, 60% are found intravitreally and 40% are subretinal.[1] In our case the cyst was lodged over the retina in Case 1, presumably having broken through the retina at that site. In Case 2 it was seen in the anterior vitreous cavity with no retinal scar. It is postulated that such a cyst could have entered the eye through the vessels of the ciliary body.[1] The cysts did not show any movement. The central scolex was neither visible clinically, nor on ultrasound B-scan or on histopathology in both the patients. The above features and the presence of inflammation suggest a dead and degenerating cyst. There are reports of dead and degenerating cysticerci causing severe inflammatory reaction and ultimately loss of the eye.[5,6] The cysts are known to release toxic products that cause severe inflammation mimicking endophthalmitis[3] and even intraocular tumor.[7]

Chronic ocular inflammation is a known etiological factor for development of rubeosis iridis and NVG.[8] Our patients may have been harboring the dead cysticercus cyst for a prolonged period causing a low-grade inflammation which eventually led to rubeosis and NVG. No visual symptoms from the cyst were noted presumably due to the location of the cyst. In Case 1 it was in the area of retina showing spontaneous reattachment and pigmentary changes. In Case 2 it was located quite anteriorly and peripherally and hence would not have caused any symptoms in a normal-sized pupil. As a result the patients presented with the symptoms of raised IOP due to NVG.

Removal of the cyst is mandatory to remove the source of the toxins causing inflammation and early removal has been advocated by many authors.[1,5]

However, both the cases also had NVG, which is known to be refractory to treatment. We are unaware of any previous reports of similar nature involving NVG due to intravitreal cysticercosis and could find no reference to it in PUBMED search. However, recently, there have been many case reports stating the efficacy of intracameral or intravitreal bevacizumab (Avastin, Genentech, Inc, San Francisco, CA) a recombinant, humanized anti-vascular endothelial growth factor (VEGF) antibody in the treatment of NVG due to other vascular disorders. It has been reported to cause rapid regression of rubeosis with a single application.[9–11] It has also been reported to improve success rate by maintaining well-functioning bleb resulting in better stabilization of IOP.[12,13] Bevacizumab is now being looked at as a promising adjunct for the treatment of NVG.

In our patients, removal of the causative agent viz. cysticercus would have resulted in the reversal of rubeosis, however, often such reversal after control of inflammation is temporary and recurrence is common.[8] Also, in advanced stage such as in Case 2 where already 270 degrees of the angle was closed causing rapid, advanced field loss it was essential to ensure good surgical success with long-term IOP control. Case 1 was one-eyed with the only seeing eye affected in this manner. This prompted us to use adjunctive bevacizumab in these two cases, to improve the immediate postoperative results. Good response was seen with no recurrence even after one year.
